# Post-COVID-19 illness and associations with sex and gender

**DOI:** 10.1186/s12872-023-03412-7

**Published:** 2023-08-08

**Authors:** Kenneth Mangion, Andrew J. Morrow, Robert Sykes, Anna Kamdar, Catherine Bagot, George Bruce, Paul Connelly, Christian Delles, Vivienne B. Gibson, Lynsey Gillespie, Pauline Hall Barrientos, Vera Lennie, Giles Roditi, Naveed Sattar, David Stobo, Sarah Allwood-Spiers, Alex McConnachie, Colin Berry, CISCO-19 investigators, Kevin G. Blyth, Michael Briscoe, Colin Church, Stephen Dobbin, Keith Gillis, Antonia Ho, David J. Lowe, Kaitlin J. Mayne, Patrick B. Mark, Christopher McGinley, Connor McKee, Oliver Peck, Alastair J. Rankin, Claire Rooney, Sarah A. Spiers, David Stobo, Ryan Wereski, Sylvia Wright, Lynn Abel, Douglas Grieve, Hannah Bayes, Jaclyn Carberry, Daniel Doherty, Ian Ferguson, Fraser Goldie, Laura Knox, Katherine Scot, David Stobo, Varun Sharma, Ammani Brown, Andrew Dougherty, Kirsty Fallon, Lesley Gilmour, Chloe Cowan, Sharon Kean, Jurgen Van-Melckebeke, Kim Moran-Jones, Debra Stuart, Maureen Travers, Tracey Hopkins, Laura Dymock, Evonne McLennan, Rosemary Woodward, Fiona Savage, Nicola Tynan, Sau Lee Chang, Mhairi Dupre, Lindsey Norton, Liam Peng, Clare Orange, Rory Gunson, Rosario Gonzalez-Lopez, Rebecca Stace, Elaine Butler, Jennifer S. Lees, Rhian M. Touyz, Paul Welsh, Massimo Palmarini, John G. F. Cleland, Sharon Kean, Bernard Kelly, Alasdair McIntosh, Dionne Russell, Sarah Weeden, Peter W. Macfarlane, Louise Inglis, Jean Watt, Kathryn McLaren, Shahid Latif, Nick Hill, Dirk Husmeier, Xiaoyu Luo, Peter Kellman, Hui Xue, Amy Collinsworth, Sarah Mullen, Campbell Rogers, Heerajnarain Bulluck, David Carrick, David Corcoran, Iain Findlay, Ninian N. Lang, Ross McGeoch, Sabrina Nordin, Alexander Payne, Keith Robertson, Nicola Ryan, Gruschen Veldtman, Robin P. Weir, Stuart Watkins, Neil Basu, Iain McInnes, Stefan Siebert

**Affiliations:** 1 School of Cardiovascular and Metabolic Health, University of Glasgow, Glasgow, UK; 2 Department of Cardiology, Queen Elizabeth University Hospital, NHS Greater Glasgow and Clyde Health Board, Glasgow, UK; 3 Department of Haemostasis and Thrombosis, Glasgow Royal Infirmary, NHS Greater Glasgow and Clyde Health Board, Glasgow, UK; 4 Department of Medical Physics, NHS G Reater Glasgow and Clyde Health Board, Glasgow, UK; 5 Project Management Unit, Glasgow Clinical Research Facility, NHS Greater Glasgow and Clyde Health Board, Glasgow, UK; 6 Department of Cardiology, Aberdeen Royal Infirmary, Aberdeen, UK; 7 Department of Radiology, NHS Greater Glasgow and Clyde Health Board, Glasgow, UK; 8 Robertson Centre for Biostatistics, School of Health and Wellbeing, University of Glasgow, Glasgow, UK

**Keywords:** COVID-19, Severe acute respiratory syndrome coronavirus-19, SARS CoV-2, Female sex, Male sex, Myocarditis, Myocardial inflammation, Post-COVID-19 syndrome

## Abstract

**Background:**

Post-COVID-19 syndromes have associated with female sex, but the pathophysiological basis is uncertain.

**Aim:**

There are sex differences in myocardial inflammation identified using cardiac magnetic resonance (CMR) in post-COVID-19 patients, and in patient reported health outcomes following COVID-19 infection.

**Design:**

This prospective study investigated the time-course of multiorgan injury in survivors of COVID-19 during convalescence.

**Methods:**

Clinical information, blood biomarkers, and patient reported outcome measures were prospectively acquired at enrolment (visit 1) and 28–60 days post-discharge (visit 2). Chest computed tomography (CT) and CMR were performed at visit 2. Follow-up was carried out for serious adverse events, including death and rehospitalization.

**Results:**

Sixty-nine (43%) of 159 patients recruited were female. During the index admission, females had a lower peak C-reactive protein (74 mg/l (21,163) versus 123 mg/l (70, 192) *p* = 0.008) and peak ferritin (229 μg/l (103, 551) versus 514 μg/l (228, 1122) *p* < 0.001). Using the Modified Lake-Louise criteria, females were more likely to have definite evidence of myocardial inflammation (54% (37/68) versus 33% (30/90) *p* = 0.003). At enrolment and 28–60 days post-discharge, enhanced illness perception, higher levels of anxiety and depression and lower predicted maximal oxygen utilization occurred more commonly in women. The mean (SD, range) duration of follow-up after hospital discharge was 450 (88) days (range 290, 627 days). Compared to men, women had lower rates of cardiovascular hospitalization (0% versus 8% (7/90); *p* = 0.018).

**Conclusions:**

Women demonstrated worse patient reported outcome measures at index admission and 28–60 days follow-up though cardiovascular hospitalization was lower.

## Introduction

Immune response to infection is sex-specific and likely to drive infection severity risk and mortality risk [1, 2]. This has been reported for the severe acute respiratory syndrome coronavirus-1 (SARS-CoV1) epidemic [3] and the middle eastern respiratory syndrome (MERS) outbreak [4]. Recently, a similar male bias towards severe disease has been reported with SARS-CoV-2 [5–8] despite similar infection rates between sexes [9]. Registry data reports that this mortality difference is only partly due to sex and gender differences in high risk characteristics [7, 8] and is independent of age [10]. Men were also more likely to have symptomatic COVID-19 illness compared with women [11].

Despite the higher morbidity and mortality in men with acute SARS-CoV-2 infection, a larger percentage of women tend to be at greater risk for long-term COVID-19 manifestations irrespective of baseline severity of disease [12, 13]. In fact, women were less likely to report a recovery back to baseline, and seven times as likely to report dyspnea, and twice as likely to report fatigue [14].

Prior studies using cardiovascular magnetic resonance imaging in COVID-19 have reported imaging features of myocardial inflammation in 27–60%, [15–18] of patients but the presence and nature of sex associations are incompletely understood.

Our hypotheses were that there are sex differences in myocardial inflammation identified using cardiac magnetic resonance (CMR) in post-COVID-19 patients scanned at 28–60 days, and in patient reported health outcomes following COVID-19 infection.

## Methods

### Design

The design and the main findings of the CISCO-19 study have been reported [19, 20]. In this manuscript the terms male and female have been used when referring to biological sex, whereas man and woman are used when referring to psychosocial gender, or when these factors are not clear.

In summary, this study involved a prospective, observational, longitudinal, cohort design to assess the time-course of multiorgan injury in survivors of COVID-19 during convalescence [19]. Clinical information, a 12-lead digital ECG, blood and urine biomarkers, and patient reported outcome measures were acquired at enrolment (visit 1) and again during convalescence, 28–60 days post-discharge (visit 2). Chest computed tomography (CT), including pulmonary and coronary angiography, and cardiac MRI were carried out at visit 2. This sex-based analysis of the primary and secondary outcomes was prespecified.

### Participant identification

Patients who received hospital care for COVID-19, with or without admission, and were alive, were prospectively screened in real time using an electronic healthcare information system (TrakCare®, InterSystems®, USA) and daily hospital reports identifying inpatients with laboratory-positive results for COVID-19.

### Eligibility criteria

The inclusion criteria were: (1) age ≥ 18 years old; (2) history of an unplanned hospital visit e.g., emergency department, or hospitalization > 24 h for COVID-19 confirmed by a polymerase chain reaction (PCR) test; (3) ability to comply with study procedures; and (4) ability to provide written informed consent. Imaging results were reported by accredited radiologists according to contemporary, national guidelines [21].

The exclusion criteria were: (1) contra-indication to magnetic resonance (MR) imaging (e.g., severe claustrophobia, metallic foreign body); and (2) lack of informed consent.

### Diagnosis of COVID-19

A diagnosis of COVID-19 was based on laboratory evidence of SARS-CoV-2 infection using a PCR test (Roche Cobas 6800 or Seegene SARS-CoV-2 PCR) on a biospecimen [22].

### Research schedule

The protocol involved two visits. The first visit involved informed consent and assessments during the initial hospitalization, or as soon as possible after discharge. The second visit occurred 28–60 days post-discharge. This window was positioned to reflect the convalescent phase and give sufficient scope to schedule the patients.

The procedures involved prospective collection of clinical data and a time-course of research investigations. Clinical data included demographics, medical and cardiovascular history, findings from clinical examinations, laboratory and radiological tests, cardiology tests (including an electrocardiogram (ECG) and an echocardiogram if available) and treatment. The research investigations at both visits included blood and urine samples, a 12-lead ECG, health status questionnaires, and assessments of adverse events. Heart, lung, and kidney imaging were acquired at the second visit.

### Biomarkers

Blood samples were collected at enrolment (visit 1) and 28–60 days post discharge (visit 2). Circulating biomarkers of cardiac injury (troponin I, N-terminal (NT)-pro hormone brain natriuretic peptide (NT-proBNP), inflammation (C-reactive protein, ferritin, interleukin-6), thrombosis (TCT ratio, D-Dimer, fibrinogen, Factor VIII, antithrombin, protein C, protein S), endothelial activation (von Willebrand factor (vWF):GP1bR, VWF:Ag, ICAM-1, VCAM-1, p-selectin, ST2) and renal function (serum creatinine, glomerular filtration rate (GFR) was estimated using the Chronic Kidney Disease Epidemiology (CKD-EPI) Equation [23]), and their changes over time, were investigated. The measurements were undertaken in a central laboratory, blinded to the other clinical data.

### Multimodality imaging

#### CT

A 320-detector CT scanner (Aquilion ONE, Canon Medical Systems Corp.) provided full heart coverage within a single heartbeat. Non-contrast and contrast-enhanced angiographic breath-hold ECG-gated volumes were acquired and timed for optimum pulmonary and systemic arterial (coronary) opacification. Patients with severe renal dysfunction underwent non-contrast CT.

#### Cardiovascular MRI

Cardiovascular MRI was undertaken to measure heart structure and function and assess for persisting evidence of myocardial injury and/or myocardial infarction using multi-parametric techniques. All patients underwent protocol-directed MRI in the convalescent phase, 28–60 days after discharge. The scan protocol included cine-imaging of cardiac anatomy and function and myocardial tissue characterization using multiparametric techniques. They included 1) mapping myocardial native longitudinal relaxation time (T1 in milliseconds) using the modified Look-Locker inversion recovery technique (T1-mapping) before and after intravenous administration of gadolinium contrast media (0.15 mmol/kg of Magnevist, Bayer Healthcare), 2) mapping transverse relaxation time (T2 in milliseconds), 3) first pass contrast-enhanced perfusion and 4) late gadolinium-enhancement imaging.

The expert consensus recommendations for the MRI diagnostic criteria of non-ischemic myocardial inflammation (modified Lake Louise criteria) were used to diagnose definite myocardial inflammation (abnormal T2 and T1 (native T1, late gadolinium enhancement or extracellular volume)) or probable myocardial inflammation (abnormal: T2 or T1) [24] To limit selection bias, patients with severe renal dysfunction (GFR < 45 ml/kg/m^2^) were not excluded but did not have gadolinium contrast. Quantitative analyses were undertaken in a central laboratory, blinded to the other clinical data.

### Health status and patient reported outcome measures

Questionnaires were completed by participants at visit 1 (enrolment) and visit 2 (28–60 days after the last episode of hospital care). Self-reported health status was assessed using EuroQOL EQ-5D-5L and the Brief Illness Perception Questionnaire (Brief-IPQ). The Patient Health Questionnaire-4 (PHQ–4) was utilized to assess for anxiety and depressive disorders. The Duke Activity Status Index (DASI) was used to assess predicted maximal oxygen utilization. The International Physical Activity Questionnaire—Short Form (IPAQ-SF) measures the forms and intensity of physical activity and sitting time that participants do as part of their daily lives.

### Longitudinal follow-up for clinical outcomes

Clinical research team members assessed study participants’ electronic health records. Serious adverse events (SAE), (comprising death and rehospitalization), outpatient clinic visits were assessed as part of follow-up.

Cardiovascular and respiratory SAE were independently reviewed and adjudicated by the clinical event committee team members.

### Statistics

Statistical analysis was carried out by the Robertson Centre for Biostatistics. Summary statistics are presented as Mean ± SD, Median (IQR), or N (%). Normally distributed continuous variables, non-normally distributed continuous variables, and categorical variables were analysed by T-Test, Wilcoxon-Mann–Whitney Test, or Fisher's Exact Test respectively.

Linear mixed effects models for patient reported outcome measures (PROMs) include random effects for subjects, fixed effects for age and sex, fixed effects for study visits, and an interaction between sex and visit. Model-derived estimates are reported for differences between women and males at each study visit, and for mean changes between study visits in women and men, with 95% confidence intervals and two-sided *p*-values. *P*-values for sex-by-visit interactions are also reported. Duration of follow-up is summarised as Median (IQR), and compared between groups using a Wilcoxon-Mann–Whitney test. Clinical outcomes by sex are summarised, and compared between groups using log rank tests of the time to first event. No adjustments were made for multiplicity, and a *p*-value of less than 0.05 was considered statistically significant.

### Trial management and timelines

The study was conducted in line with the current *Guidelines for Good Clinical Practice in Clinical Trials* and *STrengthening the Reporting of OBservational studies in Epidemiology* guidelines, and coordinated by a Study Management Group. A Scientific Steering Group had oversight of the study.

### Ethics

The study was approved by the UK National Research Ethics Service (Reference 20/NS/0066).

### Registration

ClinicalTrials.gov: NCT04403607.

## Results

One thousand three hundred and six patients were screened between 22 May 2020 and 16 March 2021 and 267 patients provided written informed consent (Cognitive impairment, *n* = 87; frailty, *n* = 341; death, *n* = 101; no consent, *n* = 356; non-compliance with protocol, *n* = 154).

One hundred and fifty-nine patients were evaluated at 28–60 days after the last episode of hospital care. Most of this population was unvaccinated. 2 (1.3%) had first dose of SARS-CoV-2 vaccination prior to admission, and 11 (6.9%) had the first dose of vaccination prior to Visit 2. Sixty-nine (43%) of them were female. Compared with males, females were younger (52.8 (12.3) years vs. 55.8 (11.5) years; *p* = 0.113) and were more often healthcare workers (26 (38%) vs. 10 (11%); *p* < 0.001). There was no difference in ethnic background (*p* = 0.641). Females had a higher body mass index (32.1 (8.7) kg/m^2^ vs. 29.2 (5.4) kg/m^2^; *p* = 0.012) and lower 10-year percentage cardiovascular risk (%, Q risk 3 calculator, https://qrisk.org/) (9.1 (9.4) vs. 16.5 (11.2); *p* < 0.001). More males had a history of hypertension (46%, 41/90 versus 22%, 15/69; *p* = 0.002), myocardial infarction (17%, 15/90 versus 3%, 2/69; *p* = 0.008) and heart failure (7%, 6/90 versus 0%; *p* = 0.036). There were no differences in other co-morbidities, presenting characteristics, or treatment (Table 1).

**Table 1:** Clinical characteristics of the study population, by sex

	All	Sex		*p*-value
		Male	Female	
	*N* = 159	*N* = 90	*N* = 69	
*Demographics*				
Age, years	54.5 ± 11.9	55.8 ± 11.5	52.8 ± 12.3	*p* = 0.113
Most deprived SIMD quintile	61 (40%)	40 (45%)	21 (33%)	*p* = 0.178
Healthcare Worker	36 (23%)	10 (11%)	26 (38%)	*p* = 0.001
Ethnicity				
*White*	139 (87%)	80 (89%)	59 (86%)	*p* = 0.641
*Asian*	14 (9%)	8 (9%)	6 (9%	
*Other*	6 (4%)	2 (2%)	4 (6%)	
*Presenting Characteristics*				
Weight, kg	87 ± 18	90 ± 17	83 ± 18	*p* = 0.012
Height, cm	169 ± 11	175 ± 8	162 ± 9	*p* < 0.001
Body mass index, kg/m^2^	30.5 ± 7.1	29.2 ± 5.4	32.1 ± 8.7	*p* = 0.012
Body surface area, m^2^	2.0 ± 0.2	2.1 ± 0.2	1.9 ± 0.2	*p* < 0.001
Heart Rate, bpm	95 ± 19	94 ± 19	96 ± 20	*p* = 0.573
Systolic blood pressure, mmHg	128 ± 20	131 ± 21	125 ± 18	*p* = 0.082
Diastolic blood pressure, mmHg	77 ± 13	79 ± 13	75 ± 12	*p* = 0.052
Peripheral oxygen saturation, %	93 ± 7	93 ± 7	94 ± 6	*p* = 0.202
Respiratory rate, /min	23 ± 12	23 ± 8	25 ± 17	*p* = 0.273
WHO Clinical severity score				
*Hospitalized, no oxygen therapy*	50 (31%)	26 (29%)	24 (35%)	*p* = 0.301
*Oxygen by mask or nasal prongs*	74 (47%)	42 (47%)	32 (46%)	
*Non-invasive ventilation*	20 (13%)	15 (17%)	5 (7%)	
*Mechanical ventilation*	15 (9%)	7 (8%)	8 (12%)	
*COVID-19 diagnosis*				
PCR test	159 (100%)	90 (100%)	69 (100%)	*p* = 1.000
Nosocomial	7 (4%)	6 (7%)	1 (1%)	*p* = 0.140
Radiology, chest radiograph or CT scan				
*Typical of COVID-19*	109 (75%)	61 (73%)	48 (76%)	*p* = 0.953
*Atypical of COVID-19*	11 (8%)	7 (8%)	4 (6%)	
*Unlikely*	4 (3%)	2 (2%)	2 (3%)	
*Normal*	22 (15%)	13 (16%)	9 (14%)	
*Acute COVID-19 therapy*				
Oxygen	109 (69%)	64 (71%)	45 (65%)	*p* = 0.492
Steroid	89 (56%)	53 (59%)	36 (52%)	*p* = 0.424
Antiviral	42 (26%)	27 (30%)	15 (22%)	*p* = 0.279
Non-invasive respiratory support	31 (19%)	22 (24%)	9 (13%)	*p* = 0.105
Intensive care	24 (15%)	14 (16%)	10 (14%)	*p* = 1.000
Invasive ventilation	14 (9%)	6 (7%)	8 (12%)	*p* = 0.398
Intravenous inotrope	7 (4%)	3 (3%)	4 (6%)	*p* = 0.469
*Cardiovascular History*				
Smoking				
*Never*	106 (67%)	60 (67%)	46 (67%)	*p* = 0.708
*Former*	44 (28%)	26 (29%)	18 (26%)	
*Current*	9 (6%)	4 (4%)	5 (7%)	
Hypercholesterolaemia	76 (48%)	48 (53%)	28 (41%)	*p* = 0.149
Hypertension	56 (35%)	41 (46%)	15 (22%)	*p* = 0.002
Diabetes mellitus	35 (22%)	22 (24%)	13 (19%)	*p* = 0.444
Chronic kidney disease	7 (4%)	5 (6%)	2 (3%)	*p* = 0.700
CCS Angina Class				
*No Angina*	154 (97%)	86 (96%)	68 (99%)	*p* = 0.389
*Angina Class I-IV*	5 (3%)	4 (4%)	1 (1%)	
Heart failure	6 (4%)	6 (7%)	0 (0%)	*p* = 0.036
Myocardial infarction	17 (11%)	15 (17%)	2 (3%)	*p* = 0.008
Stroke or TIA	5 (3%)	3 (3%)	2 (3%)	*p* = 1.000
Peripheral vascular disease	1 (1%)	1 (1%)	0 (0%)	*p* = 1.000
Previous PCI	10 (6%)	9 (10%)	1 (1%)	*p* = 0.044
Previous CABG	2 (1%)	2 (2%)	0 (0%)	*p* = 0.506
Cardiovascular disease and/or treatment	74 (47%)	49 (54%)	25 (36%)	*p* = 0.026
*Risk Scores*				
ISARIC-4c in-hospital mortality risk, in %	12.1 ± 10.6	14.8 ± 11.1	8.5 ± 8.6	*p* = 0.001
Q-Risk 3, 10-year cardiovascular risk, in %	13.5 ± 11.1	16.5 ± 11.2	9.1 ± 9.4	*p* = 0.001
Charlson Comorbidity Index	1.9 ± 1.8	2.1 ± 2.0	1.6 ± 1.6	*p* = 0.131
*Pre-existing maintenance medication*				
Aspirin	12 (8%)	10 (11%)	2 (3%)	*p* = 0.067
Statin	46 (29%)	32 (36%)	14 (20%)	*p* = 0.052
Beta-blocker	20 (13%)	15 (17%)	5 (7%)	*p* = 0.093
Angiotensin converting enzyme inhibitor	35 (22%)	26 (29%)	9 (13%)	*p* = 0.020
Angiotensin receptor blocker	10 (6%)	6 (7%)	4 (6%)	*p* = 1.000
Oral anticoagulation	8 (5%)	3 (3%)	5 (7%)	*p* = 0.295
*Laboratory results, index admission*				
Initial haemoglobin, g/l	141 ± 16	146 ± 14	134 ± 16	*p* < 0.001
Initial platelet count, 10^9^/l	237 ± 94	238 ± 108	236 ± 72	*p* = 0.847
Initial white cell count, 10^9^/l	7.29 ± 5.52	7.95 ± 6.84	6.42 ± 2.87	*p* = 0.083
Initial lymphocyte count, 10^9^/l	1.53 ± 4.66	1.76 ± 6.18	1.23 ± 0.63	*p* = 0.482
Peak D-Dimer, ng/ml	1740 ± 5493	1822 ± 5935	1633 ± 4923	*p* = 0.867
Minimum eGFR, ml/min/1.73m^2^	81.6 ± 27.4	81.1 ± 26.2	82.4 ± 29.1	*p* = 0.771
Acute kidney injury	20 (14%)	11 (13%)	9 (15%)	*p* = 0.809
Peak hs-troponin I, ng/l	4.0 (3.0, 12.8)	5.0 (3.5, 14.5)	4.0 (2.0, 8.0)	*p* = 0.039
Peak ferritin, μg/l	360 (180, 864)	514 (228, 1122)	229 (103, 551)	*p* < 0.001
Peak C-reactive protein, mg/l	104 (37, 181)	123 (70, 192)	74 (21, 163)	*p* = 0.008
Peak HbA1c, mmol/mol	48.0 ± 18.4	50.0 ± 19.9	45.3 ± 15.9	*p* = 0.143
Initial albumin, g/l	34.2 ± 5.2	34.1 ± 5.1	34.2 ± 5.4	*p* = 0.842
*Timelines*				
Hospitalised	143 (90%)	83 (92%)	60 (87%)	*p* = 0.298
Duration of admission, days	5 (3, 10)	6 (3, 10)	5 (2, 12)	*p* = 0.434
Symptom onset to primary outcome, days	64 (53, 72)	62 (52, 72)	66 (55, 72)	*p* = 0.464
Diagnosis to primary outcome, days	61 (49, 69)	58 (48, 69)	61 (51, 68)	*p* = 0.587

Summaries are Mean ± SD, Median (IQR), or N (%). *P*-values from T-Test, Wilcoxon-Mann–Whitney Test, or Fisher's Exact Test

### Multisystem phenotyping and adjudicated myocarditis

#### Biochemistry

During the index admission, females had a lower peak C-reactive protein (74 mg/l (21,163) versus 123 mg/l (70, 192) *p* = 0.008) and peak ferritin (229 μg/l (103, 551) versus 514 μg/l (228, 1122) *p* < 0.001).

#### Electrocardiology

There were no differences in ECG criteria by sex (Table 2).

**Table 2:** Multisystem phenotypying by sex: serial electrocardiography, biomarkers of inflammation, metabolism, renal function, and haemostasis, and heart, lung, and kidney imaging at 28–60 days post-discharge

	All	Sex		*p*-value
		Male	Female	
*Electrocardiogram*				
*Myopericarditis criteria*				
Admission	*N* = 150	*N* = 83	*N* = 67	
	31 (21%)	19 (23%)	12 (18%)	*p* = 0.545
Enrolment	*N* = 147	*N* = 82	*N* = 65	
	47 (32%)	27 (33%)	20 (31%)	*p* = 0.859
28–60 days post-discharge	*N* = 143	*N* = 83	*N* = 60	
	33 (23%)	20 (24%)	13 (22%)	*p* = 0.841
*CT Chest 28–60 days post-discharge*				
	*N* = 157	*N* = 90	*N* = 67	
Ground glass opacity and/or consolidation	70 (45%)	43 (48%)	27 (40%)	*p* = 0.418
Reticulation and/or architectural distortion	47 (30%)	29 (32%)	18 (27%)	*p* = 0.487
Atelectasis	13 (8%)	9 (10%)	4 (6%)	*p* = 0.400
Pulmonary arterial thrombus	5 (3%)	2 (2%)	3 (5%)	*p* = 0.650
Visual estimate of % of total lung area abnormal	14.3 ± 19.0	15.8 ± 19.7	12.3 ± 18.0	*p* = 0.252
*CT coronary angiogram 28–60 days post-discharge*				
	*N* = 156	*N* = 89	*N* = 67	
Coronary calcium—Agatston score	144 ± 502	216 ± 651	50 ± 124	*p* = 0.042
MESA percentile	59.4 ± 34.7	59.5 ± 30.2	59.4 ± 41.1	*p* = 0.997
Obstructive coronary artery disease	21 (14%)	17 (19%)	4 (6%)	*p* = 0.031
*FFR*_*CT*_* patient-level (all coronary arteries) 28–60 days post-discharge*				
	*N* = 132	*N* = 75	*N* = 57	
Median FFR_CT_	0.93 ± 0.03	0.92 ± 0.04	0.93 ± 0.03	*p* = 0.078
Minimum FFR_CT_	0.80 ± 0.10	0.80 ± 0.10	0.80 ± 0.10	*p* = 0.982
Minimum FFR_CT_ ≤ 0.8	50 (38%)	27 (36%)	23 (40%)	*p* = 0.718
*Cardiac MRI 28–60 days post-discharge*				
	*N* = 159	*N* = 90	*N* = 69	
LV end diastolic volume index, ml/m^2^	75.9 ± 17.0	82.1 ± 16.3	67.9 ± 14.3	*p* < 0.001
LV end systolic volume index, ml/m^2^	35.3 ± 12.8	40.1 ± 13.9	28.9 ± 7.4	*p* < 0.001
LV ejection fraction, %	54.1 ± 9.7	51.8 ± 9.7	57.2 ± 8.8	*p* < 0.001
LV mass, g	91.8 ± 25.6	104.7 ± 23.0	75.1 ± 18.0	*p* < 0.0001
RV end diastolic volume index, ml/m^2^	73.3 ± 17.7	80.4 ± 16.9	64.3 ± 14.5	*p* < 0.0001
RV end systolic volume index, ml/m^2^	35.9 ± 11.3	40.3 ± 11.1	30.3 ± 8.9	*p* < 0.0001
RV ejection fraction, %	50.9 ± 10.5	49.6 ± 9.8	52.6 ± 11.1	*p* = 0.076
*Myocardial tissue characterisation*				
	*N* = 158	*N* = 90	*N* = 68	
Increased global T1 (> 1233 ms)	55 (35%)	21 (23%)	34 (50%)	*p* < 0.001
Increased global T2 (> 44 ms)	10 (6%)	4 (4%)	6 (9%)	*p* = 0.330
T2 ratio (myocardium/serratus anterior muscle)	1.69 ± 0.23	1.66 ± 0.20	1.73 ± 0.25	*p* = 0.072
Increased global extracellular volume (> 27.4%)	71 (50%)	31 (38%)	40 (65%)	*p* = 0.002
*Late gadolinium enhancement*				
	*N* = 158	*N* = 90	*N* = 68	
Myocardial late gadolinium enhancement	30 (19%)	27 (30%)	3 (4%)	*p* < 0.001
Ischaemic distribution	8 (6%)	7 (9%)	1 (2%)	*p* = 0.138
Non-ischaemic distribution	24 (16%)	22 (27%)	2 (3%)	*p* < 0.001
Myocardial inflammation (Lake Louise criteria)				
*No evidence (0/2)*	17 (11%)	15 (17%)	2 (3%)	*p* = 0.003
*Probable (1/2)*	74 (47%)	45 (50%)	29 (43%)	
*Definite (2/2)*	67 (42%)	30 (33%)	37 (54%)	
*Biomarkers at enrolment, central laboratory*				
	*N* = 156	*N* = 89	*N* = 67	
eGFR, ml/min/1.73m^2^	96 (85, 105)	96 (85, 106)	95 (85, 105)	*p* = 0.764
C-reactive protein, mg/l	5.5 (1.6, 22.3)	6.0 (1.7, 26.4)	5.3 (1.5, 12.1)	*p* = 0.355
hs-troponin I, ng/l	3.3 (2.2, 5.8)	4.0 (2.7, 7.0)	2.7 (1.9, 4.2)	*p* < 0.001
NT pro BNP, ng/l	114 (57, 262)	99 (51, 285)	120 (63, 250)	*p* = 0.518
Total bilirubin, μmol/l	5.7 (4.3, 7.9)	6.2 (4.8, 8.8)	5.0 (3.5, 6.7)	*p* < 0.001
Total Cholesterol, mmol/l	4.83 ± 1.38	4.53 ± 1.39	5.22 ± 1.26	*p* = 0.002
Triglycerides, mmol/l	2.25 ± 1.25	2.20 ± 1.24	2.31 ± 1.28	*p* = 0.604
HDL Cholesterol, mmol/l	1.06 ± 0.33	0.96 ± 0.27	1.20 ± 0.36	*p* < 0.001
*Biomarkers at 28–60 days post-discharge, central laboratory (control group samples from enrolment visit)*				
	*N* = 158	*N* = 90	*N* = 68	
eGFR, ml/min/1.73m^2^	95 (83, 106)	96 (81, 105)	94 (86, 106)	*p* = 0.924
C-reactive protein, mg/l	1.9 (0.9, 3.5)	1.7 (0.7, 3.2)	2.2 (1.2, 4.4)	*p* = 0.056
hs-troponin I, ng/l	2.1 (1.3, 4.0)	3.2 (1.6, 5.3)	1.6 (0.6, 2.2)	*p* < 0.001
NT pro BNP, ng/l	83 (54, 198)	78 (52, 192)	100 (63, 196)	*p* = 0.439
D-Dimer, ng/ml	205 ± 252	214 ± 289	195 ± 198	*p* = 0.645

Summaries are Mean ± SD, Median (IQR), or N (%). *P*-values from T-Test, Wilcoxon-Mann–Whitney Test, or Fisher's Exact Test

#### CT chest, coronary and pulmonary angiography

There were no differences in pulmonary parameters by sex (Table 2). Women were less likely to have obstructive coronary artery disease (6% (4/67) versus 19% (17/90), *p* = 0.031.

#### Cardiovascular magnetic resonance imaging

Differences in volumes in keeping with accepted sex specific ranges were observed in ventricular dimensions [25]. Females had higher global T1 values compared to males (50%, 34/68 versus 23%, 21/90; *p* < 0.001) and increased global extracellular volume values (65% 40/68 versus 38%, 31/90; *p* = 0.002).

Females had less myocardial late gadolinium enhancement in a non-ischemic distribution, when compared to males (3% (2/68) versus 27% (22/90); *p* < 0.001).

Using the Modified Lake-Louise criteria [24], females were more likely to have definite evidence of myocardial inflammation (54% (37/68) versus 33% (30/90) *p* = 0.003).

#### Health status

Compared to men, at enrolment and 28–60 days post-discharge, women demonstrated enhanced illness perception, higher levels of anxiety and depression and lower predicted maximal oxygen utilization (ml/kg/min) (Table 3). Furthermore, at 28–60 days post-discharge women had lower health-related quality of life, and lower levels of physical activity (Table 3).

**Table 3:** Health status, illness perception, anxiety and depression, and physical function by sex

	All	Sex		*p*-value
		Male	Female	
Enrolment	*N* = 153	*N* = 86	*N* = 67	
28–60 days post-discharge	*N* = 158	*N* = 90	*N* = 68	
*Health-related Quality of Life, EQ-5D-5L*				
Heath Utility Score at enrolment	0.74 ± 0.22	0.77 ± 0.23	0.71 ± 0.19	*p* = 0.072
Heath Utility Score at 28–60 days post-discharge	0.77 ± 0.23	0.82 ± 0.20	0.71 ± 0.26	*p* = 0.003
Your Health Today VAS at enrolment	61.46 ± 21.89	65.22 ± 21.66	56.64 ± 21.40	*p* = 0.016
Your Health Today VAS at 28–60 days post-discharge	72.64 ± 19.60	75.18 ± 18.12	69.32 ± 21.06	*p* = 0.063
*Brief Illness Perception Questionnaire Score*				
At enrolment	42.4 ± 12.3	40.3 ± 12.8	45.0 ± 11.2	*p* = 0.019
At 28–60 days post-discharge	36.5 ± 14.7	33.0 ± 14.2	41.2 ± 14.0	*p* < 0.001
Anxiety score at enrolment	2.13 ± 2.08	1.76 ± 1.96	2.62 ± 2.15	*p* = 0.012
Anxiety score at 28–60 days post-discharge	1.81 ± 2.00	1.40 ± 1.72	2.37 ± 2.23	*p* = 0.003
Depression score at enrolment	2.19 ± 1.95	1.84 ± 1.83	2.65 ± 2.02	*p* = 0.010
Depression score at 28–60 days post-discharge	1.78 ± 1.90	1.48 ± 1.71	2.18 ± 2.09	*p* = 0.023
Total score at enrolment	4.32 ± 3.78	3.59 ± 3.61	5.27 ± 3.82	*p* = 0.006
Total score at 28–60 days post-discharge	3.59 ± 3.71	2.89 ± 3.19	4.55 ± 4.16	*p* = 0.006
*Physical Function*				
*IPAQ score at enrolment*				
*Low*	112 (80%)	61 (77%)	51 (84%)	*p* = 0.665
*Moderate*	16 (11%)	10 (13%)	6 (10%)	
*High*	12 (9%)	8 (10%)	4 (7%)	
*IPAQ score at 28–60 days post-discharge*				
*Low*	68 (52%)	30 (38%)	38 (73%)	*p* < 0.001
*Moderate*	44 (34%)	35 (44%)	9 (17%)	
*High*	19 (15%)	14 (18%)	5 (10%)	
DASI score at enrolment	19.6 ± 18.0	23.7 ± 19.9	14.4 ± 13.8	*p* = 0.002
DASI score at 28–60 days post-discharge	24.2 ± 17.6	29.0 ± 18.7	17.9 ± 13.9	*p* < 0.001
DASI VO_2_ max estimate at enrolment	18.0 ± 7.8	19.8 ± 8.6	15.8 ± 5.9	*p* = 0.002
DASI VO_2_ max estimate at 28–60 days post-discharge	20.0 ± 7.6	22.1 ± 8.1	17.3 ± 6.0	*p* < 0.001

Summaries are Mean ± SD, Median (IQR), or N (%). *P*-values from T-Test, Wilcoxon-Mann–Whitney Test, or Fisher's Exact Test

Adjusting for age and study visits, health-related quality of life, enhanced illness perception, higher levels of anxiety and depression, lower levels of physical activity and lower predicted maximal oxygen utilization were evident in women (Table 4).

**Table 4:** Linear mixed effects regression models for patient reported outcomes in relation to sex

	Estimate (95% CI)	*p*-value	Interaction *p*-value
EQ-5D VAS			
Sex difference (F-M) at enrolment	-7.0 (-12.2, -1.8)	*p* = 0.008	*p* = 0.557
Sex difference (F-M) at 28–60 days post-discharge	-5.2 (-11.0, 0.5)	*p* = 0.073	
Change from enrolment to 28–60 days (Female)	13.3 (8.9, 17.8)	*p* < 0.001	
Change from enrolment to 28–60 days (Male)	11.6 (7.7, 15.4)	*p* < 0.001	
EQ-5D Health Utility			
Sex difference (F-M) at enrolment	-0.047 (-0.106, 0.012)	*p* = 0.116	*p* = 0.280
Sex difference (F-M) at 28–60 days post-discharge	-0.083 (-0.148, -0.017)	*p* = 0.013	
Change from enrolment to 28–60 days (Female)	0.038 (-0.010, 0.086)	*p* = 0.122	
Change from enrolment to 28–60 days (Male)	0.073 (0.031, 0.116)	*p* < 0.001	
Brief Illness Perception Questionnaire Score			
Sex difference (F-M) at enrolment	2.18 (-1.29, 5.65)	*p* = 0.218	*p* = 0.008
Sex difference (F-M) at 28–60 days post-discharge	7.32 (3.50, 11.15)	*p* < 0.001	
Change from enrolment to 28–60 days (Female)	-2.28 (-5.12, 0.56)	*p* = 0.115	
Change from enrolment to 28–60 days (Male)	-7.42 (-9.91, -4.94)	*p* < 0.001	
PHQ4 Total Score			
Sex difference (F-M) at enrolment	0.71 (-0.25, 1.68)	*p* = 0.148	*p* = 0.233
Sex difference (F-M) at 28–60 days post-discharge	1.36 (0.28, 2.44)	*p* = 0.013	
Change from enrolment to 28–60 days (Female)	-0.44 (-1.25, 0.37)	*p* = 0.283	
Change from enrolment to 28–60 days (Male)	-1.09 (-1.78, -0.39)	*p* = 0.002	
PHQ4 Anxiety Score			
Sex difference (F-M) at enrolment	0.34 (-0.19, 0.87)	*p* = 0.208	*p* = 0.097
Sex difference (F-M) at 28–60 days post-discharge	0.84 (0.25, 1.43)	*p* = 0.005	
Change from enrolment to 28–60 days (Female)	-0.05 (-0.50, 0.39)	*p* = 0.810	
Change from enrolment to 28–60 days (Male)	-0.55 (-0.94, -0.17)	*p* = 0.005	
PHQ4 Depression Score			
Sex difference (F-M) at enrolment	0.37 (-0.13, 0.87)	*p* = 0.144	*p* = 0.548
Sex difference (F-M) at 28–60 days post-discharge	0.55 (-0.01, 1.11)	*p* = 0.053	
Change from enrolment to 28–60 days (Female)	-0.38 (-0.83, 0.06)	*p* = 0.093	
Change from enrolment to 28–60 days (Male)	-0.56 (-0.94, -0.18)	*p* = 0.004	
IPAQ High Physical Activity (differences reported as odds ratios)			
Sex difference (F-M) at enrolment	0.66 (0.03, 15.93)	*p* = 0.799	*p* = 0.779
Sex difference (F-M) at 28–60 days post-discharge	1.00 (0.12, 8.49)	*p* = 0.999	
Change from enrolment to 28–60 days (Female)	12.79 (1.00, 163.08)	*p* = 0.050	
Change from enrolment to 28–60 days (Male)	8.44 (1.42, 50.19)	*p* = 0.019	
IPAQ Moderate/High Physical Activity (differences reported as odds ratios)			
Sex difference (F-M) at enrolment	0.76 (0.36, 1.59)	*p* = 0.465	*p* = 0.1870
Sex difference (F-M) at 28–60 days post-discharge	0.41 (0.20, 0.83)	*p* = 0.014	
Change from enrolment to 28–60 days (Female)	1.23 (0.59, 2.55)	*p* = 0.578	
Change from enrolment to 28–60 days (Male)	2.29 (1.29, 4.05)	*p* = 0.005	
DASI Score			
Sex difference (F-M) at enrolment	-6.78 (-11.19, -2.37)	*p* = 0.003	*p* = 0.271
Sex difference (F-M) at 28–60 days post-discharge	-9.54 (-14.39, -4.69)	*p* < 0.001	
Change from enrolment to 28–60 days (Female)	4.11 (0.39, 7.82)	*p* = 0.030	
Change from enrolment to 28–60 days (Male)	6.86 (3.61, 10.12)	*p* < 0.001	

Models include random effects for subjects, fixed effects for age and sex, fixed effects for study visits, and an interaction between sex and visit. Model-derived estimates reported for difference between women and males at each study visit, and for mean changes between study visits in women and men. *P*-values for sex-by-visit interactions also reported

#### Serious adverse events

Follow-up was continued to December 13, 2021 for all participants. The mean (SD, range) duration of follow-up after hospital discharge was 450 (88) days (range 290, 627 days).

Compared to men, women had lower rates of cardiovascular hospitalization (0% versus 8% (7/90); *p* = 0.018)(Table 5).

**Table 5:** Clinical outcomes by sex. Duration of follow-up summarised as Median (IQR), and compared between groups using a Wilcoxon-Mann–Whitney test

	All	Sex		*p*-value
		Male	Female	
	*N* = 159	*N* = 90	*N* = 69	
*Duration of Follow-up*				
Days to Visit 3 or death	419 (369, 451)	418 (380, 444)	421 (369, 468)	*p* = 0.569
*Outcomes*				
Death or Hospitalization (Any Cause)	24 (15%)	15 (17%)	9 (13%)	*p* = 0.566
Death (Any Cause)	2 (1%)	1 (1%)	1 (1%)	*p* = 0.865
Cardiovascular Death	1 (1%)	0 (0%)	1 (1%)	*p* = 0.266
Renal Death	0 (0%)	0 (0%)	0 (0%)	-
Respiratory Death	0 (0%)	0 (0%)	0 (0%)	-
Hospitalization (Any Cause)	24 (15%)	15 (17%)	9 (13%)	*p* = 0.566
Cardiovascular Hospitalization	7 (4%)	7 (8%)	0 (0%)	*p* = 0.018
Renal Hospitalization	2 (1%)	1 (1%)	1 (1%)	*p* = 0.980
Respiratory Hospitalization	8 (5%)	2 (2%)	6 (9%)	*p* = 0.066
*Cardiovascular Outcomes*				
Myocardial infarction	1 (1%)	1 (1%)	0 (0%)	*p* = 0.379
Percutaneous Coronary Intervention	3 (2%)	3 (3%)	0 (0%)	*p* = 0.126
Coronary Artery Bypass Grafting	0 (0%)	0 (0%)	0 (0%)	-
Cerebrovascular accident	0 (0%)	0 (0%)	0 (0%)	-
Heart Failure	2 (1%)	2 (2%)	0 (0%)	*p* = 0.214
Deep vein thrombosis	0 (0%)	0 (0%)	0 (0%)	-
New atrial fibrillation	3 (2%)	3 (3%)	0 (0%)	*p* = 0.125
Ventricular tachycardia or fibrillation	0 (0%)	0 (0%)	0 (0%)	-
*Respiratory Outcomes*				
Pulmonary fibrosis	9 (6%)	3 (3%)	6 (9%)	*p* = 0.146
New diagnosis asthma	1 (1%)	0 (0%)	1 (1%)	*p* = 0.253
Pulmonary embolism	4 (3%)	1 (1%)	3 (4%)	*p* = 0.197
Long-term oxygen therapy	2 (1%)	0 (0%)	2 (3%)	*p* = 0.105
*Secondary Care (Outpatients)*				
Any Outpatient Referral	108 (68%)	58 (64%)	50 (72%)	*p* = 0.504
Acute COVID-19 (< 28 days)	15 (9%)	9 (10%)	6 (9%)	*p* = 0.799
Ongoing COVID-19 (28–84 days)	20 (13%)	10 (11%)	10 (14%)	*p* = 0.479
Long COVID-19 (> 84 days)	58 (36%)	29 (32%)	29 (42%)	*p* = 0.387
Cardiology	22 (14%)	15 (17%)	7 (10%)	*p* = 0.222
Respiratory	55 (35%)	29 (32%)	26 (38%)	*p* = 0.590
Physiotherapy	22 (14%)	12 (13%)	10 (14%)	*p* = 0.885

Clinical outcomes summarised as number and percentage with at least one event, and compared between groups using log rank test of time to first event

## Discussion

This study assessed sex and gender differences in a deeply phenotyped cohort of patients utilizing serum and urine biochemistry, patient reported outcomes and electrocardiograms at baseline and 28–60 days after hospital discharge; cross sectional imaging with computed tomography and magnetic resonance imaging and clinical follow-up up to a mean of 450 days after hospital discharge.

The main findings were:

1. No difference in COVID-19 illness severity between women and men with regards to length of stay or therapy (including intensive care).
2. Male sex was associated with history of hypertension, myocardial infarction and heart failure
3. Female sex was associated with lower peak markers of inflammation during their admission (ferritin, C-reactive protein)
4. There was no sex difference in myopericarditis criteria on ECG.
5. Female sex was associated with lower rates of obstructive coronary artery disease on computed tomography.
6. Female sex was associated with higher cardiac T1 and extracellular volume fraction values. Female sex was associated with higher rates of definitive myocardial inflammation utilizing the Modified Lake-Louise criteria.
7. Worse patient reported outcome measures at index admission and 28–60 days follow-up were demonstrated in women compared to men.
8. Female sex was associated with lower rates of hospitalization for cardiovascular disease in the longer term after COVID-19 infection.

Unlike the meta-analysis reported by Peckham et al. [9], where men had higher odds of requiring intensive care admission or death, we did not identify a difference in acute disease severity for women and men in our smaller study. However, we report persisting changes identified on magnetic resonance imaging and patient reported outcome measures associated with female sex. These findings complement data reported by Bai et al. [12] Sylvester et al. [13] and others that women tend to be at higher risk for COVID-19 manifestations persisting in the longer term irrespective of baseline severity of disease [12, 13].

Women had lower rates of hypertension, myocardial infarction or heart failure, and no difference in other past medical history parameters commonly reported as being associated with worse outcomes in COVID-19 infection including diabetes or smoking status [26]. There was no sex difference in various pulmonary computed tomographic parameters including consolidation, reticulation, atelectasis or thrombosis. A retrospective study looking at 1165 patients reported that female sex was associated with less severe lung involvement [27]. These differences could be due to study design (prospective versus retrospective) and different inclusion criteria (all hospitalized patients) and data analyses (lung involvement quantified utilizing an AI system [28]).

In this study, female sex was associated with lower peak markers of inflammation when compared with male sex, consistent with data reported from the MGH COVID-19 patient registry (*n* = 781) [29]. These observations point to an immunological basis in the different course of the disease according to sex as per Brodin [30].

To date, there are no reports describing sex- and/or gender- specific differences in myocardial injury utilizing cardiac magnetic resonance imaging in COVID-19 patients in the acute or longer-term [15–18]. Whilst elevated T1 values *per se* may be a non-specific finding on its own (elevated in patients with hypertensive heart disease, diabetes), when analyzed as part of the modified Lake-Louise criteria [24] i.e. having both a positive T2-based marker and a T1-based marker (T1 value (ms), extra cellular volume fraction, myocardial late gadolinium enhancement) will increase specificity for diagnosing acute myocardial inflammation, having only one (T2 or T1) marker may still support a diagnosis of acute myocardial inflammation in an appropriate clinical scenario, albeit with less specificity.

A number of studies looking at both hospitalized and non-hospitalized patients [13, 31–33] with COVID-19 reported higher rates of depression, anxiety and illness perception in women participants.

There were similar rates of outpatient healthcare uptake between men and women, and similar rates of serious adverse events (death or rehospitalization). In our study, female sex was associated with lower rates of hospitalization for cardiovascular disease in the longer term after COVID-19 infection.

The apparent discrepancy between higher rates of patient reported outcomes in women and increased hospitalization for cardiovascular causes in men could be explained by the small sample size in our study (possible type 1 error), the higher, pre-exisiting cardiovascular disease burden in men, or the possibility that the hospitalization events for cardiovascular causes were not related to COVID-19 infection.

Imaging was carried out from day 28 post discharge to align with the International Severe Acute Respiratory and Emerging Infection Coronavirus Clinical Characterisation Consortium (ISARIC4C) study [34]. Most of the study participants were unvaccinated and the results cannot be extrapolated to vaccinated individuals.

## Conclusion

Despite there being no difference in COVID-19 illness severity between women and men with regards to length of stay or therapy, female sex was associated with lower peak markers of inflammation during hospital admission, worse patient reported outcome measures at index admission and 28–60 days follow-up though cardiovascular hospitalization was lower.

Female sex was associated with higher rates of definitive myocardial inflammation on CMR utilizing the Modified Lake-Louise criteria.

## Data Availability

The datasets used and/or analysed during the current study available from the corresponding author on reasonable request. Data requests will be considered by the Steering Group which includes representatives of the Sponsor, the University of Glasgow, senior investigators independent of the research team, and the chief investigator. The Steering Group will take account of the scientific rationale, ethics, logistics, and resource implications. Data access requests should be initially submitted by email to the Chief Investigator (Colin Berry, corresponding author). The source data includes the deidentified numerical data used for the statistical analyses and deidentified imaging scans (MRI, CT) and ECGs. Data access will be provided through the secure analytical platform of the Robertson Centre for Biostatistics. This secure platform enables access to deidentified data for analytical purposes, without the possibility of removing the data from the server. Requests for transfer of deidentified data (including source imaging scans) will be considered by the Steering Group and if approved, a collaboration agreement would be expected. The Steering Group will consider any cost implications and cost recovery would be expected on a not-for-profit basis.
